# Insulin secretory actions of ethanolic extract of *Acacia arabica* bark in high fat-fed diet-induced obese Type 2 diabetic rats

**DOI:** 10.1042/BSR20230329

**Published:** 2023-05-23

**Authors:** Prawej Ansari, Sara S. Islam, Samia Akther, Joyeeta T. Khan, Jaber A. Shihab, Yasser H. A. Abdel-Wahab

**Affiliations:** 1 Department of Pharmacy, School of Pharmacy and Public Health, Independent University, Bangladesh (IUB), Dhaka 1229, Bangladesh; 2 Centre for Diabetes, School of Biomedical Sciences, Ulster University, Coleraine BT52 1SA, U.K.

**Keywords:** GLP-1, glucose, Insulin, obesity, phytoconstituents, type 2 diabetes

## Abstract

*Acacia arabica* commonly known as ‘babul’ has been widely used for the treatment of numerous diseases, including diabetes due to their potential pharmacological actions. The aim of the present study was to investigate the insulinotropic and antidiabetic properties of ethanol extract of *Acacia arabica* (EEAA) bark through *in vitro* and *in vivo* studies in high fat-fed (HFF) rats. EEAA at 40–5000 µg/ml significantly increased (*P*<0.05–0.001) insulin secretion with 5.6 and 16.7 mM glucose, respectively, from clonal pancreatic BRIN BD11 β-cells. Similarly, EEAA at 10–40 µg/ml demonstrated a substantial (*P*<0.05–0.001) insulin secretory effect with 16.7 mM glucose from isolated mouse islets, with a magnitude comparable to 1 µM glucagon-like peptide-1 (GLP-1). Diazoxide, verapamil, and calcium-free conditions decreased insulin secretion by 25–26%. The insulin secretory effect was further potentiated (*P*<0.05–0.01) with 200 µM isobutylmethylxanthine (IBMX; 1.5-fold), 200 µM tolbutamide (1.4-fold), and 30 mM KCl (1.4-fold). EEAA at 40 µg/ml, induced membrane depolarization and elevated intracellular Ca^2+^ as well as increased (*P*<0.05–0.001) glucose uptake in 3T3L1 cells and inhibited starch digestion, glucose diffusion, dipeptidyl peptidase-IV (DPP-IV) enzyme activity, and protein glycation by 15–38%, 11–29%, 15–64%, and 21–38% (*P*<0.05, 0.001), respectively. In HFF rats, EEAA (250 mg/5 ml/kg) improved glucose tolerance, plasma insulin, and GLP-1 levels, and lowered DPP-IV enzyme activity. Phytochemical screening of EEAA revealed the presence of flavonoids, tannins and anthraquinone. These naturally occurring phytoconstituents may contribute to the potential antidiabetic actions of EEAA. Thus, our finding suggests that EEAA, as a good source of antidiabetic constituents, would be beneficial for Type 2 diabetes patients.

## Introduction

Diabetes-related mortality has alarmingly escalated in response to the rising prevalence of the disease globally [1]. The initial manifestation of diabetes is the impediment to glucose uptake in muscles due to insulin resistance, which causes excessive blood glucose and abnormal accumulation at various cellular locations leading to hyperglycaemia [2]. Inadequate insulin production and impaired muscle glucose uptake result in significantly critical complications such as nephropathy, retinopathy, and neuropathy, as well as the production of superoxide free radicals due to hyperglycaemia-induced protein glycation [3,4]. Additionally, the correlation between obesity and diabetes has an effect on several organ systems and is linked to various cardiovascular diseases [5]. The development of atherosclerosis and a high mortality rate, as found in Type 2 diabetic patients, are particularly linked with an altered lipid profile or dyslipidemia [6]. It has been found that 90% of individuals with Type 2 diabetes are considered to be overweight or obese, making obesity a major variable risk factor for the development of Type 2 diabetes [5].

Proper nutrition, weight maintenance, and regular physical activity are necessary to keep glycaemic levels under control [7]. In addition, single or combined synthetic oral antidiabetic medicines such as biguanides, sulfonylureas, dipeptidyl peptidase-IV (DPP-IV) inhibitors, thiazolidinediones, disaccharidase inhibitors, glucagon-like peptide-1 (GLP-1) and glucose-dependent insulinotropic polypeptide (GIP) analogs, or/and insulin can be employed as insulin secretagogues/insulinotropic agents for the management of diabetes mellitus [8]. These synthetic drugs, however, present various adverse effects including hypoglycemia, weight gain, gastrointestinal tract (GIT) disorders, hypersensitivity reactions, liver, and kidney diseases, and are often unavailable and inaccessible to people residing in rural areas [9–11]. Therefore, to overcome the drawbacks of synthetic medicines, it is crucial to search for alternative medications that are mostly derived from natural sources such as medicinal plants and animal derived peptides [9].

From the beginning of civilization, medicinal plants have been considered an excellent source of therapeutics owing to their plethora of health benefits. Herbal medicines have long been used to cure a wide range of ailments due to the presence of numerous bioactive phytoconstituents that exhibit various pharmacological actions, and their proven efficacy, lower incidence of adverse effects in clinical studies, and affordability have encouraged many medical professionals to practice them in practical life [9,12]. Over 12,000 species of medicinal plants have been identified to exhibit insulin-releasing and glucose-lowering action [12]. The majority of these plants contain several classes of phytoconstituents such as flavonoids, alkaloids, carotenoids, terpenoids, steroids, tannins, saponins, phenolic acids, and glycosides [13,14]. The antidiabetic activity of these compounds is generally attributed to improvement in pancreatic β-cell function by increasing insulin secretion, decreasing intestinal glucose absorption, or facilitating metabolism [15]. Thus, pure compounds of medicinal plants or their crude extracts can be formulated as dietary supplements or antidiabetic therapy to aid in the treatment of diabetes mellitus.

*Acacia arabica*, popularly known as Babul, is a tree belonging to the family of *Leguminosae* and has been used in traditional medical practice for centuries [14]. Nearly all of its parts including the bark, gum, leaves, roots, flowers, and pods are used as medicines. *Acacia arabica* is well known around the world as a multipurpose tree and is used to treat bleeding disorders, prolapse, leucorrhea, gastrointestinal disorder, diarrhea, constipation, and diabetes in traditional medical practice [16]. In *Ayurvedic* medicine, the gum of *Acacia arabica* is extensively utilized as a dietary supplement to manage diabetes [13]. Pharmacological studies have shown that *Acacia arabica* has antioxidant, antidiabetic, antihypertensive, antispasmodic, antibacterial, and antifungal properties [16]. A recent study conducted on obese high-fat fed rats indicated that the hot water extract of *Acacia arabica* inhibits glucose absorption, DPP-IV enzyme activity and improves β-cell function [13]. However, although *Acacia arabica* is considered to exert glucose-lowering effects, only a few studies have been conducted to assess its impact on insulin secretion and action [17]. Thus, the current experiment was carried out to investigate the *in vitro* and *in vivo* antidiabetic effects of ethanol extract of *Acacia arabica* (EEAA) bark to elucidate its mode of action in the management of Type 2 diabetes.

## Materials and methods

### Collection and preparation of plant extracts

*Acacia arabica* bark was collected and identified by a taxonomist at the Bangladesh National Herbarium and assigned the accession number 43756 [13]. The obtained barks were rinsed, air-dried, and then ground to a fine powder. The dry powdered (200 g) bark was macerated in 1 L of 80% ethanol and agitated in an orbital shaker at a speed of 550 rpm for 48–72 h. The filtrate was separated using Whatman no.1 filter paper and then dried off using a rotary evaporator (BibbyRE-200, Sterilin Ltd., Newport, U.K.) machine. The gummy residue was freeze-dried in a freeze-dryer (Varian 801 LY-3-TT, Lexington, MA, U.S.A.) and preserved at 4°C until further assayed [8].

### *In vitro* insulin-release studies using BRIN-BD11 cells

Clonal pancreatic BRIN-BD11 β-cells were used for examining the insulin-releasing effects of EEAA *in vitro*. EEAA or insulin modulators in the presence or absence of glucose (5.6, or 16.7 mM) were incubated with BRIN-BD11 cells in a CO_2_ incubator at 37°C for 20 min. The effects of EEAA in the presence of insulin secretagogues or inhibitors, such as tolbutamide (a sulphonylurea and K_ATP_ channel blocker), diazoxide (a K_ATP_ channel opener), verapamil (a voltage-dependent Ca^2+^ channel blocker), IBMX (a phosphodiesterase inhibitor), 30 mM KCl, and 10 mM alanine, were studied in order to determine the insulin-releasing pathways activated by EEAA. Membrane depolarization and Ca^2+^ influx is induced by KCl and alanine. Alanine mostly accomplishes this via co-transport with Na^+^ and metabolism with the generation of ATP [18,19].

### Insulin-release studies using isolated mouse islets

The impact of EEAA on insulin release was also investigated by using isolated mouse islets. Pancreatic tissue of mice (40–50 g, b.w.) was digested with collagenase P obtained from *Clostridium histolyticum* (Sigma-Aldrich, Dorset, U.K.) to extract the islets. Islets were cultured for 48–72 h in a CO_2_ incubator at 37°C. Further islets were incubated with 1.4 and 16.7 mM glucose for 1 h, with or without EEAA, alanine, and GLP-1, respectively. Centrifugation was used to separate the supernatants, which were stored at −20°C for radioimmunoassay to measure the insulin concentration [20]. An acid–ethanol extraction method was employed to measure the insulin content of islet cells [21].

### Membrane potential and intracellular calcium ion ([Ca^2+^]i) concentration

We used a Fluorometric Imaging Plate Reader (FLIPR) Membrane Potential and [Ca^2+^]i assay kit (Molecular Devices, Sunnyvale, CA, U.S.A.) to determine the intensity of membrane depolarization and [Ca^2+^]i in BRIN-BD11 cells treated with the EEAA. BRIN-BD11 cells were seeded in 96-well plates and kept overnight in a CO_2_ incubator at 37°C for adherence. After the medium was withdrawn, the cells were pre-incubated with 100 μl of 5.6 mM glucose KRB buffer at 37°C for 10 min. Following the addition of 100 μl of FLIPR membrane potential or calcium dye, the cells were incubated at 37°C for 60 min. FlexStation 3 (Molecular Devices, Sunnyvale, CA, U.S.A.) was used to measure the fluctuations in signal intensity. Depolarising concentrations of KCl (30 mM) and alanine (10 mM) were employed as positive controls [8,13].

### Glucose uptake

The effect of EEAA on cellular glucose uptake was assessed using adipocytes produced from 3T3L1 fibroblast cells. The cells were treated with the EEAA and kept in a CO_2_ incubator at 37°C for half an hour in the presence or absence of 100 nM insulin. The incubation was continued with 2-(N-(7-Nitrobenz-2-oxa-1,3-diazol-4-yl)Amino)-2-Deoxyglucose, 2-NBDG (50 nM) for an additional 5 min. Coverslips were fixed to the slides after the cells were rinsed with ice-cold PBS. Using a fluorescent microscope (10× magnification), magnified images of the fluorescence intensity were taken to evaluate the glucose uptake [13,19].

### Glycation of insulin

The impact of EEAA on insulin glycation was examined as previously described [22]. To conduct the experiment, D-glucose (246.5 mM) was incubated with human insulin (1 mg/ml) and NaBH_3_CN (0.0853 g/ml) with or without the EEAA treatment. After 24 h of incubation, the reaction was stopped by the addition of 0.5 M acetic acid. Measurement of glycated and non-glycated insulin were completed using reverse-phase HPLC [8].

### DPP-IV enzyme activity *in vitro*

Using a fluorometric technique, the effects of EEAA on the DPP-IV enzyme activity were studied *in vitro*. The 96-well black-walled, clear-bottomed Greiner microplates containing 8 mU/ml of DPP-IV enzyme and 200 µM of Gly-Pro-AMC substrate were used to measure the enzyme activity as previously described [23]. Variations in fluorescence were monitored using the Flex Station 3 (Molecular Devices) with excitation and emission wavelengths at 370 and 440 nm with a 2.5 nm slit width, respectively [24].

### Starch digestion

To investigate the impact on starch digestion, EEAA or acarbose was incubated with 100 mg of starch solution (Sigma-Aldrich, St. Louis, MO, U.S.A.). After dilution, the mixture was treated with thermostable α-amylase (0.01%) from *Bacillus leicheniformis* and amyloglucosidase (0.1%) from Rhizopus mold (Sigma-Aldrich, St. Louis, U.S.A.). The GOD-PAP method (Randox GL 2623) was implemented to further analyze the final samples aliquoted for the measurement of glucose concentration [8].

### Glucose diffusion *in vitro*

A cellulose ester dialysis tube (CEDT) (20 cm 7.5 mm, Spectra/Por®CE layer, MWCO: 2000, Spectrum, The Netherlands) was used to test the effects of EEAA on glucose diffusion *in vitro*. To perform the experiment, the tubes were filled with 2 ml of 0.9% NaCl and 220 mM glucose in the presence or absence of the EEAA, and the ends were tightly sealed. Afterward, the CEDT was put into 50 ml Falcon conical tubes (Orange Scientific, Orange, CA, U.S.A.) containing 0.9% NaCl (45 ml) solution and agitated in an orbital shaker at 20 ± 2°C. The samples were collected 24 h later, and the GOD-PAP method (Randox GL 2623), as previously reported [18,25], was used to analyze the aliquoted samples for the detection of glucose diffusion and absorption.

### Animals

The experiments were carried out on 6–8 weeks aged male Sprague Dawley rats (Envigo, U.K.) weighing between 200 and 250 g. Prior to the experiments, the animals were given access to a high-fat diet for 6–8 weeks consisting of 20% protein, 45% fat, and 35% carbohydrates with a total energy content of 26.15 kJ/g. A standard diet of 10% fat, 30% protein, and 60% carbohydrates with a metabolizable energy content of 12.99 kJ/g was fed to normal rats (Trouw Nutrition, Cheshire, U.K.). The animals were accommodated in regulated conditions of 25 ± 0.5°C temperature and 65–70% humidity and an automated 12 h dark–light cycle system was installed in the animal house to maintain a day–night circadian rhythm. Before performing the experiments, the fasting blood glucose was determined in HFF diet rats in order to distinguish each group. HFF diet-induced obese Type 2 diabetic rats were defined as those with fasting blood glucose levels that were higher than normal (>6.0 mmol/L). The groups were divided in the following manner:

Group 1: Lean control (Saline)
Group 2: High fat-fed diet control (Saline)
Group 3: High fat-fed diet + EEAA (250 mg/5 ml/kg)
Group 4: High fat-fed diet + sitagliptin (10 µmol/5 ml/kg)
Group 5: High fat-fed diet + vildagliptin (10 µmol/5 ml/kg)

### Oral glucose tolerance

To assess the effects of EEAA on oral glucose tolerance, the high fat-fed (HFF) rats were starved overnight and oral gavage of glucose (18 mmol/kg, body weight [b.w.]) with or without the treatment (250 mg/5 ml/kg, b.w.) were given to both normal and HFF rats. Samples of blood were obtained using heparinized microvessel blood collection tubes (Sarstedt, Numbrecht, Germany) from the tip of the tail at 0 min before and at 30, 60, 120, and 180 min after the glucose/drug administration. Followed by centrifugation at 12,000 rpm at 4°C for 5 min, the plasma was separated and stored at −20°C until further insulin assay. Blood glucose levels were measured using Ascencia Contour glucose meters (Bayer, Newbury, U.K.) and insulin levels were measured by a dextran-charcoal radioimmunoassay [8,13].

### DPP-IV enzyme activity *in vivo*

A fluorometric assay was employed to study the impact of EEAA on plasma DPP-IV enzyme activity in HFF rats. Blood samples were taken from overnight fasted HFF rats before (at 0 min) and after (30, 60, 120, and 180 min) oral administration of EEAA (250 mg/5 ml/kg), the DPP-IV inhibitors, vildagliptin (10 µmol/5 ml/kg), and sitagliptin (10 µmol/5 ml/kg) or saline (5 ml/kg). Plasma serum was separated by centrifugation and the samples (10 μl) were incubated in 96-well microplates with 40 μl of Tris-HCl (100 mM) buffer (pH 7.4) and 50 μL of Gly-Pro-AMC (200 μM) substrate for 30 min at 37°C. Hydrolysis of the fluorogenic substrate bonds (H-Gly-Pro) conjugated to the AMC group (H-Gly-Pro-AMC) by the DPP-IV enzyme in the blood serum caused the formation of the fluorescent 7-amino-4-methyl coumarin (AMC). As mentioned above in the section on *in vitro* DPP-IV enzyme activity, the fluorescence changes were monitored using FlexStation 3. Plasma samples collected at 60 min were used to determine levels of active GLP-1 (7-36) using a GLP-1 (Active) ELISA Kit (EGLP-35K, Merck Millipore, Dorset, U.K.) [21].

### Phytochemical screening

The EEAA was subjected to phytochemical screening to determine the presence or absence of phytochemicals including glycosides, reducing sugars, flavonoids, alkaloids, terpenoids, tannins, and anthraquinones as per previous methods [21,26–28].

#### Alkaloids

Alkaloid testing was done by acidifying 2 ml of the EEAA in dilute hydrochloric acid to which 1 ml of Dragendroff’s reagent was added. The precipitate's color change from orange to crimson red confirmed the presence of alkaloids [26].

#### Flavonoids

The presence of flavonoids was tested by mixing 4 ml of the EEAA with 1.5 ml of methanol, which was then heated. Upon the addition of magnesium metal together with 2–3 drops of hydrochloric acid, the solution's color changed to pink indicating a positive result [26].

#### Tannins

To test for tannins, a few drops of 10% lead acetate were added to 2 ml of the EEAA. The formation of white sediment suggested the presence of tannins [21].

#### Terpenoids

Terpenoids were tested by dissolving 1 g of the EEAA in 2 ml of chloroform to which 3mL of strong sulphuric acid was carefully added to form a layer; the presence of terpenoids was indicated by a reddish-brown coloration on the interface [27].

#### Glycosides

To test for glycosides, 1 ml of the EEAA was combined with a few drops of glacial acetic acid, and ferric chloride to form a mixture, to which concentrated sulfuric acid was added afterward. The presence of glycoside was evidenced by the visualization of a blue-green color [21].

#### Anthraquinone

To test for anthraquinones, a dry test tube was filled with about 0.5 g of the EEAA, 5 ml of chloroform, and was shaken vigorously for 5 min. After filtering the mixture, an equal amount of 10% ammonia solution was mixed into the filtrate and the presence of anthraquinone was confirmed upon the formation of pink-violet or red color in the lower layer [28].

#### Reducing sugars

Reducing sugars were detected by mixing 1 ml of the EEAA, 1 ml of distilled water, and a few (4–6) drops of Fehling’s reagent, and the mixture was heated. The formation of a reddish-brown color confirmed the presence of reducing sugars [21].

### Statistical analysis

All statistical analysis and data interpretation were conducted using GraphPad Prism 5. The unpaired Student’s *t*-test (nonparametric, with two-tailed *P*-values) and one-way or two-way ANOVA with Sidek *post-hoc* tests were used to analyze the data. The significance threshold was set at *P*<0.05, and values were presented as the mean ± SEM.

## Results

### EEAA and insulin release from BRIN-BD11 cells

**Figure 1:**
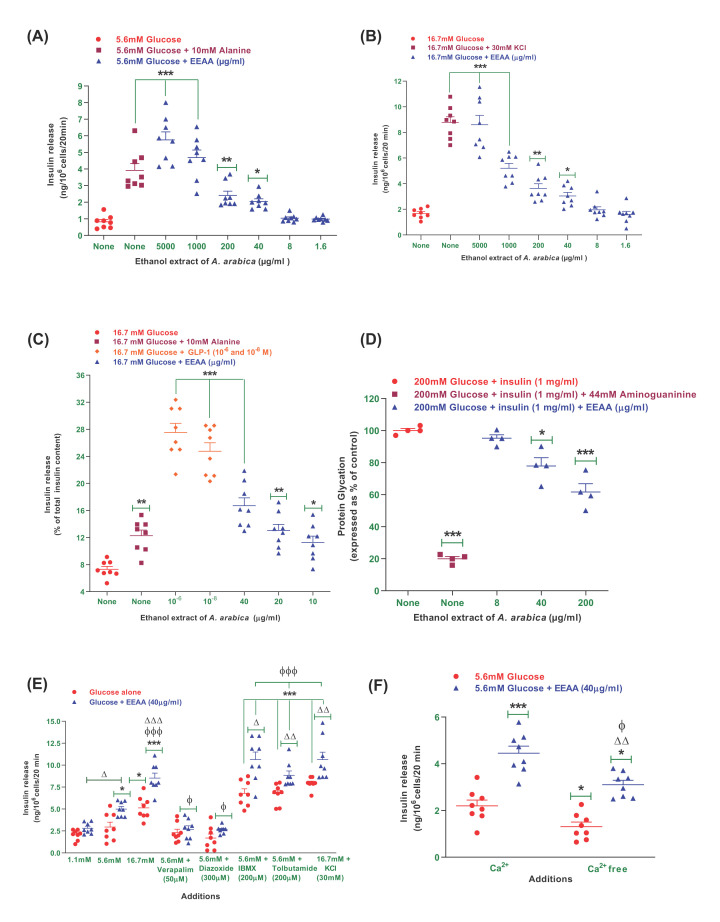
Effects of ethanol extract of *Acacia arabica* (EEAA) bark on insulin secretion from (A,B) clonal pancreatic BRIN BD11 β cells, as well as (C) pancreatic islets of Langerhans, (D) protein glycation, (E) insulin secretion with known stimulators or inhibitors and (F) with or without extracellular calcium Values are mean ± SEM; *n* = 4–8 for insulin secretion and glycation of protein. ^*, **, ***^P<0.05–0.001 compared with control. ^ϕ^*P*<0.05 and ^ϕϕϕ^*P*<0.001 compared with 5.6 mM glucose with EEAA. ^Δ, ΔΔ, ΔΔΔ^*P*<0.05–0.001 compared with respective incubation without EEAA. EEAA, ethanol extract of *Acacia arabica* (bark).

Concentration-dependent (1.6–5000 µg/ml) insulin-releasing effects of EEAA are presented in Figure 1A,B. The basal rate of insulin release at 5.6 mM glucose (Figure 1A) from BRIN-BD11 cells was 0.89 ± 0.02 ng/10^6^ cells/20 min. The positive control, alanine (10 mM) increased the insulin-releasing rate to 4.45 ± 0.54 ng/10^6^ cells/20 min (Figure 1A; *P*<0.001; *n*=8). EEAA (40–5000 µg/ml) increased insulin release from 2.05 ± 0.21 to 7.1 ± 1.1 ng/10^6^ cells/20 min (Figure 1A; *P*<0.05–0.001) with 5.6 mM glucose. In the presence of 16.7 mM glucose (Figure 1B), the basal insulin rate was 1.53 ± 0.12 ng/10^6^ cells/20 min and with the depolarising concentration of KCl (30 mM), it was increased to 8.78 ± 0.69 ng/10^6^ cells/20 min (*P*<0.001). Additionally, EEAA with 16.7 mM glucose enhanced the release of insulin from 3.05 ± 0.31 to 8.86 ± 1.58 ng/10^6^ cells/20 min (Figure 1B; *P*<0.05–0.001) in a dose-dependent manner (40–5000 µg/ml). An increase in LDH release was observed with increasing extract concentrations, however, there was no effect on cellular viability at lower doses (data not shown).

### EEAA and insulin release from isolated mouse islets

The insulin-releasing effects of EEAA from isolated mouse islets are illustrated in Figure 1C. At 16.7 mM, the basal rate of insulin secretion from isolated mouse islets was 7.15 ± 0.78 ng/10^6^ cells/20 min. EEAA showed a significant increase (Figure 1C; *P*<0.05–0.001) in insulin secretion from 11.29 ± 1.02 to 16.71 ± 1.24 with 16.7 mM glucose in a concentration-dependent manner (10–40 µg/ml). As a positive control alanine (10 mM) and GLP-1 (10^−6^ and 10^−8^ M) significantly stimulated (Figure 1C; *P*<0.001) the release of insulin from 12.27 ± 0.94 to 27.53 ± 1.42 at 16.7 mM glucose. However, the increase in insulin secretion by EEAA was lower than the GLP-1 (10^−6^ and 10^−8^ M) in presence of 16.7 mM glucose.

### EEAA and known modulators of insulin release and, inhibitors or absence of extracellular calcium

EEAA (40 µg/ml) bark was treated with established insulin releasing modulators to assess their insulin secretory actions (Figure 1E). The release of insulin was significantly augmented (Figure 1E) with modulators such as 16.7 mM glucose (*P*<0.05), IBMX (*P*<0.001), and tolbutamide (*P*<0.001). EEAA resulted in a significant rise in insulin secretion by 1.4-fold when combined with a depolarizing concentration of 30 mM KCl (*P*<0.01; Figure 1E). Insulin release activity was further increased following co-treatment of EEAA with IBMX (by 1.5-fold; *P*<0.05) and tolbutamide (by 1.3-fold; *P*<0.01). In the presence of K^+^ channel activator diazoxide (300 µM), L-type voltage-dependent Ca^2+^ channels blocker verapamil (50 µM), and free extracellular Ca^2+^, the insulin-releasing rate was attenuated by 25–26%, respectively (Figure 1E,F).

### EEAA and membrane depolarization and, [Ca^2+^]_i_ in BRIN-BD11 cells

**Figure 2:**
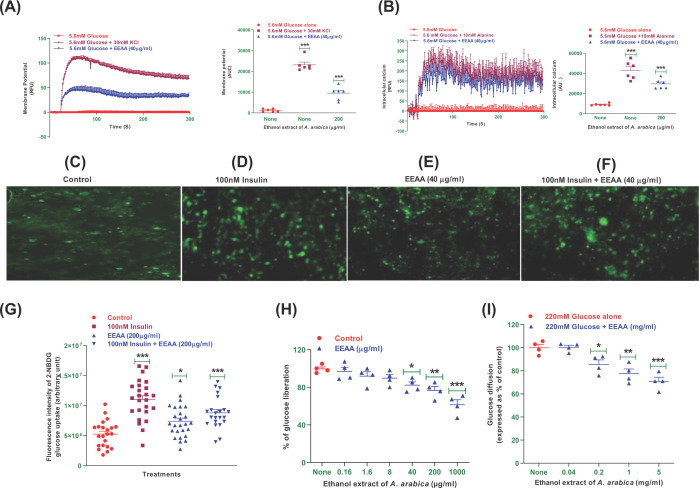
Effects of ethanol extract of *Acacia arabica* (EEAA) bark on (A) membrane potential and (B) intracellular calcium in clonal pancreatic BRIN-BD11 β-cells and, (C–G) glucose uptake by differentiated 3T3L1 adipocytes, (H) starch digestion and (I) glucose diffusion *in* *vitro* The intensity of fluorescence was measured for cells incubated with EEAA (E) minus or (F) plus 100 nM insulin. The images were captured at 10x magnification. (G) Glucose uptake in 3T3L1 cells and percentage of glucose liberation from (H) starch digestion and (I) glucose diffusion *in vitro* were represented in the scatter dot plot. The values are mean ± SEM; *n* = 6 for membrane potential and intracellular calcium, *n* = 4 for glucose uptake, starch digestion and glucose diffusion. ^*, **, ***^P<0.05–0.001 compared with control.

Depolarization of membrane potential and intracellular calcium ([Ca^2+^]_i_) concentration in clonal BRIN-BD11 cells were assessed (Figure 2A,B). A significant induce in membrane depolarization (94%; Figure 2A) and an increase in intracellular calcium concentration ([Ca^2+^]_i_) (80%; Figure 2B) were observed in incubation with KCl (30 mM) and alanine (10 mM). EEAA at a concentration of 40 µg/ml induced (*P*<0.001) depolarization of membrane potential by 87% (Figure 2A) followed by an increase in [Ca^2+^]_i_ concentration by 69% (Figure 2B).

### EEAA and glucose uptake and insulin action

The glucose analogue 2-(N-(7-Nitrobenz-2-oxa-1,3-diazol-4-yl) Amino)-2-Deoxyglucose (2-NBDG) fluorescent hexose was used to assess the effect of EEAA on glucose uptake and insulin action using 3T3L1 differentiated adipocyte cells (Figure 2C–G). In the microscopic fluorescence analysis, EEAA enhanced glucose uptake significantly with (*P*<0.05; Figure 2G) or without (*P*<0.001; Figure 2G) insulin (100 nM) when compared with the control. Insulin alone stimulated glucose uptake by 2.6-fold (*P*<0.001; Figure 2G) compared with the control.

### EEAA and glycation of insulin

EEAA showed a significant inhibitory effect on insulin glycation (Figure 1D). EEAA caused a 21.6% inhibition at the concentration of 40 µg/ml (*P*<0.05; Figure 1D), while the effect increased to 38.4% at 200 µg/ml (*P*<0.001; Figure 1D). With 44 mM aminoguanidine, the inhibition of insulin glycation increased to 80% (*P*<0.001; Figure 1D).

### EEAA and starch digestion

The effects of EEAA on starch digestion are shown in Figure 2H. Acarbose (1 mg/ml), employed as a positive control, decreased enzymatic glucose liberation from starch by 72% (data not shown). EEAA at a dose of 40–1000 µg/ml showed 14–38% inhibitory activity (*P*<0.05–0.001) in glucose liberation from starch.

### EEAA and glucose diffusion *in vitro*

EEAA showed significant inhibitory effects on glucose diffusion and absorption (Figure 2I) over a 24 h incubation period compared to the control group. Doses of 0.2–5 mg/ml EEAA showed an 11–29% inhibitory effect (*P*<0.05–0.001; Figure 2I).

### EEAA and DPP-IV enzyme activity *in vitro*

**Figure 3:**
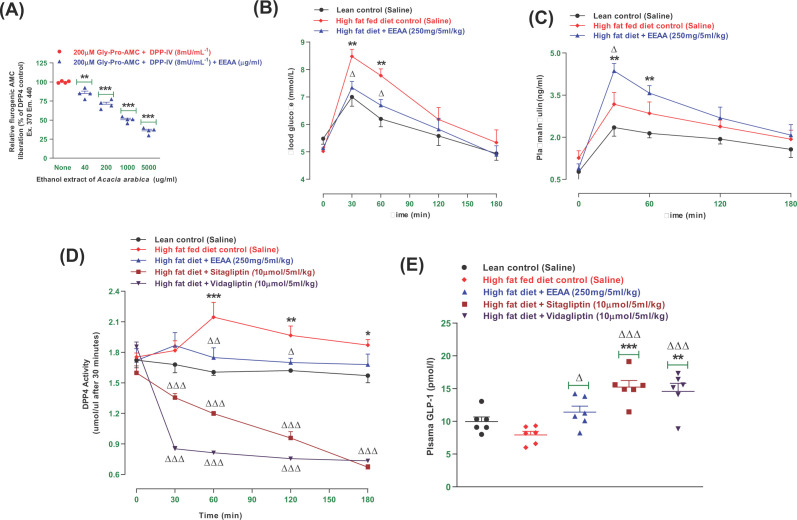
Effects of ethanol extract of *Acacia arabica* (EEAA) bark on (A) *in vitro* dipeptidyl peptidase-4 (DPP-IV) enzyme, (B) glucose tolerance, (C) plasma insulin, (D) plasma DPP-IV and (E) active GLP-1 (7-36) in HFF rats Parameters were measured before and after oral administration of glucose alone (18 mmol/kg body weight, control) or with EEAA (250 mg/5 ml/kg body weight), sitagliptin and vidagliptin (both at 10 μmol/5 ml/kg, body weight) *in vivo*. Plasma active GLP-1 (7-36) levels were evaluated at 60 min following treatment. Values are mean ± SEM; *n*=4 for *in vitro* DPP-IV enzyme activity and *n*=6, for *in vivo* parameters. ^*, **, ***^P<0.05–0.001 compared with control and ^Δ, ΔΔ, ΔΔΔ^*P*<0.05–0.001 compared with HFF diet control rats.

Effects of EEAA on DPP-IV enzyme activity (Figure 3A) were evaluated by an *in vitro* fluorometric method. An established DPP-IV inhibitor, sitagliptin (10 µM) reduced DPP-IV enzyme activity by 97.5% (data not shown). At a dose of 40–5000 µg/ml EEAA, the inhibition of the DPP-IV enzyme increased by 15–64% (*P*<0.01–0.001; Figure 3A).

### EEAA and oral glucose tolerance and plasma insulin

Oral administration of EEAA (250 mg/5 ml/kg; body weight) in conjunction with glucose (18 mmol/5 ml/kg; body weight) showed a significant (*P*<0.05; Figure 3B) reduction in blood glucose at 30 and 60 min compared with the HFF diet control. EEAA also increased plasma insulin concentrations at 30 min (*P*<0.05; Figure 3C) as compared with HFF rats.

### EEAA and DPP-IV enzyme activity and active GLP-1 (7-36) levels

EEAA (250 mg/5 ml/kg; body weight) reduced DPP-IV enzyme activity (*P*<0.05–0.01; Figure 3D) at 60 and 120 min compared with the HFF diet control group. The standard drugs sitagliptin and vildagliptin (10 μmol/5 ml /kg), showed consistent (*P*<0.001; Figure 3D) reduction in DPP-IV enzyme activity with respect to time (30–180 min). Oral gavage of EEAA (250 mg/5 ml/kg; body weight) increased the level of plasma active GLP-1 (7-36) in the circulation by 28% (*P*<0.05; Figure 3E) and this was increased to 83–92% (*P*<0.001; Figure 3E) with sitagliptin (10 µmol/5 ml/kg) and vildagliptin (10 µmol/5 ml/kg), respectively.

### Phytochemical screening of EEAA

To determine the presence of anticipated anti-diabetic phytochemicals, additional studies were done (Table 1). Flavonoids, tannins, terpenoids, glycosides, and anthraquinone were identified in EEAA (Table 1).

**Table 1:** Preliminary phytochemical analysis of ethanol extract of *Acacia arabica* bark

Group	Result
Alkaloids	−
Flavonoids	+
Tannins	+
Terpenoids	+
Glycosides	+
Anthraquinone	+
Reducing sugars	−

The ‘+’ sign denotes the presence of phytoconstituents whereas ‘−’ sign denotes the absence of phytoconstituents in EEAA. The tests were carried out three times (*n*=3).

## Discussion

The gum of *Acacia arabica* is widely employed as an ethnomedicine due to its numerous therapeutic benefits including glucose-lowering effects and has been scientifically proven to possess anti-hyperglycaemic properties [29]. Previous studies reported that hot water extract of *Acacia arabica* improves β-cell functions in HFF diabetic animal models [13]. However, the molecular mechanism underlying EEAA’s antidiabetic and insulinotropic activities has yet to be detailed [30,31]. The objective of the present study was to investigate insulin-releasing and glucose-lowering actions of EEAA through *in vitro* and *in vivo* studies to explore its underlying mechanism of action for the treatment of Type 2 diabetes.

In the present study, the insulinotropic effects of EEAA were explored using clonal pancreatic BRIN-BD11 cells and isolated mouse islets where EEAA stimulated insulin release in a dose-dependent manner. The mechanisms underlying the stimulation of insulin secretion were also investigated using non-toxic concentrations of EEAA in the presence or absence of known modulators of β-cell function. In response to 16.7mM glucose, EEAA stimulated basal insulin secretion. The effects of tolbutamide and membrane-depolarizing concentrations of KCl (30 mM) were examined to evaluate their effects in the absence and presence of EEAA. It is known that the action of this sulphonylurea involves the closure of K_ATP_ channels, depolarization of the plasma membrane, and stimulation of Ca^2+^ influx via the activation of L-type voltage-dependent calcium channels [32]. In both conditions, EEAA increased insulin release indicating the ability of EEAA to potentiate insulin secretion through various mechanistic pathways including a direct effect on exocytosis or phosphatidylinositol (PI3) or adenylate cyclase/cAMP pathway [33]. Additionally, the stimulatory activity of EEAA also showed the involvement of ion channels in clonal pancreatic β-cells. The insulin-releasing actions of EEAA were suppressed by the K_ATP_-channel opener diazoxide, indicating that the closure of K_ATP_ channels contributes to EEAA’s insulinotropic action. These findings are consistent with our observations using the L-type voltage-dependent Ca^2+^ channel blocker, verapamil, which partially decreased EEAA-mediated insulin release, suggesting its dependency on insulin release on the Ca^2+^ channel [34]. Examining the effects of Ca^2+^ free buffer revealed a similar dependency on extracellular Ca^2+^. The effects of the absence of Ca^2+^ on insulin secretion were not fully abolished suggesting that EEAA is capable of both inducing intracellular Ca^2+^ mobilization and Ca^2+^ entry. The direct observation of intracellular Ca^2+^ in BRIN-BD11 cells also provided strong evidence for this finding. Furthermore, the phosphodiesterase inhibitor, IBMX, also potentiated the insulin-releasing effects of EEAA, indicating the involvement of the cAMP pathway [35].

Insulin plays a key role in the regulation of glucose disposal in peripheral tissues like skeletal muscles, adipose tissues, and the liver [36]. Recent studies have shown that the stimulation of glucose uptake by insulin via the insulin receptor substrate 1/phosphoinositol 3-kinase (IRS-1/P3K) and GLUT4 translocation by muscular contraction or exercise by the activation of AMPK is mediated by a distinctive intracellular signaling pathway [37]. Furthermore, as skeletal muscle is the main location for using both glucose and fatty acids, insulin resistance associated with Type 2 diabetes is mostly found in this tissue [37]. In this present study, we investigated EEAA's effects on glucose uptake in 3T3L1 adipocyte cells. It has been observed that EEAA increases glucose uptake in 3T3L1 cells. Earlier investigations on *Acacia arabica* showed the presence of kaempferol, quercetin, and gallic acids, which stimulate AMP-activated protein kinase activity and increase GLUT4 translocation [38,39]. A previous study has also demonstrated that quercetin promotes GLUT4 translocation by concurrently increasing the phosphorylation of both AMPK and AKT which in turn resulted in the stimulation of glucose uptake in skeletal muscle cells and adipose tissues [37,40]. Therefore, the presence of flavonoids such as quercetin in EEAA may be responsible for EEAA's potential to increase glucose transport in skeletal muscles and adipocyte cells via activating signaling pathways [13].

Non-enzymatic glycosylation of structural proteins is speculated to be an important factor contributing to the onset of diabetes associated complications [41]. It has been observed that the glycation of insulin decreases its biological action. This reduced biological activity of glycated insulin may be caused due to its decreased affinity for the insulin receptor, poor insulin signaling, or it may function as a ligand for the receptor for advanced glycation end products, activating oxidative stress and pro-inflammatory pathways that result in insulin resistance [42]. In our study, EEAA was found to decrease insulin glycation in a concentration-dependent manner. In earlier investigations, *Acacia arabica* was reported to contain well-known antioxidant constituents such as flavonoids, glycosides, quercetin, and gallic acids [43]. Thus, the antiglycation effects demonstrated by the EEAA may be due to its phytochemicals and antioxidant properties [44].

The effects of EEAA on α-amylase and α-glucosidase enzymes on glucose release from starch following digestion were studied *in vitro*. Acarbose, an established α-glucosidase inhibitor decreased glucose liberation significantly. The concentration-dependent inhibition of glucose release from starch was observed with EEAA. Previous studies found that flavonoids are very effective in reducing the α-amylase activity and slowing down starch digestion [45]. It is also known that increased intake of dietary fiber helps to suppress appetite. In addition, dietary fiber impedes stomach emptying and/or delays energy and nutrient absorption which results in lower post-prandial glucose and lipid levels [46]. Results from previous investigations have reported the high fibre content [47] in *Acacia arabica*, which may also be responsible for the postprandial glucose-lowering effects of EEAA due to slower digestion and longer duration of nutritional absorption.

Several medicinal plants have been found to limit gastrointestinal glucose absorption, which may be a factor in how effective they are at preventing hyperglycaemia [48]. There are different mechanisms by which medicinal plants interfere with glucose absorption into cells such as by decreasing the gastric emptying time and obstructing the absorption of glucose from the intestine, inhibiting disaccharidase enzymes like α-amylase and α-glucosidase and preventing the breakdown of carbohydrates, stimulating insulin release, inhibiting gluconeogenesis or by enhancing the uptake of glucose into peripheral cells [49]. In this present study, a simple *in vitro* dialysis-based model was utilized to examine the effects of EEAA on glucose diffusion. Although this model used constant agitation to simulate gastrointestinal movement, it has certain limitations because it does not directly compare the timing of cellular mechanisms for glucose absorption in the gut with the time it takes for glucose to completely diffuse from the dialysis tube (22–26 h). Our results have depicted that EEAA demonstrates significant dose-dependent inhibition of the movement of glucose through the dialysis membrane. These findings are in agreement with previous findings conducted on alloxan-induced diabetic rats and rabbits which reported that a diet containing *Acacia arabica* exhibits antihyperglycemic activity [50,51].

The progression of Type 2 diabetes is linked to obesity. Obesity is characterized by the presence of non-esterified fatty acids (NEFAs) released from adipose tissue, which contributes to insulin resistance and β-cell dysfunction, resulting in Type 2 diabetes. In our present study, EEAA improved glucose tolerance and plasma insulin significantly in HFF diet-induced obese rats. It was observed that the tannins present in *Acacia arabica* improves the release of insulin from pancreatic β-cells and restored their functionality [17]. Furthermore, flavonoids such as quercetin, catechin, and kaempferol were also reported to increase insulin secretion and improve glucose uptake, plasma insulin responses, and glucose tolerance in mice [9,13]. Therefore, it may be reasonable to assume that the anti-hyperglycaemic effects of EEAA are attributable to these phytomolecules.

Several pharmaceutical methods have been developed to treat Type 2 diabetes by focusing on the development of oral DPP-IV inhibitors to block the degradation of the incretin hormones GLP-1 and GIP [52]. In the current study, EEAA inhibited DPP-IV enzyme activity *in vitro* in a concentration-dependent manner, which was consistent with our *in vivo* findings in HFF rats. GLP-1 and GIP hormones play important roles in regulating insulin secretion and management of Type 2 diabetes by augmenting glucose-stimulated insulin secretion via the cAMP signaling pathway [53,54]. The combined effect of GLP-1 and GIP in stimulating insulin secretion in a glucose-dependent manner, prolonging stomach emptying time, and suppressing hunger, thus, significantly improving the management of postprandial hyperglycaemia in particular [55]. EEAA also increased the levels of active GLP-1 (7-36) in the bloodstream. Previous studies have shown that reduced levels of the antagonistic metabolite and increased amounts of active GLP-1 can be achieved by inhibiting DPP-IV enzyme activity, which could be beneficial in the treatment of impaired glucose tolerance and Type 2 diabetes. [56]. The flavonoids present in natural products and crude herbal extracts have previously been found to exert promising DPP-IV enzyme inhibitory action which acts via binding to the DPP-IV and causing a conformational shift that inhibits the active site of the enzyme [57,58]. Therefore, it may be reasonable to assume that the presence of flavonoids in EEAA may be responsible for the DPP-IV enzyme inhibition and enhancement of GLP-1 action to aid in the maintenance of glucose homeostasis.

Phytochemical screening of EEAA identified the presence of different classes of phytochemicals including tannins, terpenoids, glycosides, anthraquinones, and flavonoids such as kaempferol and quercetin [13] which are consistent with the results of earlier studies [14]. Flavonoids have previously been observed to improve glucose homeostasis and β-cell function in STZ-induced rats [59,60]. It has also been documented that the antidiabetic effect of flavonoids aid in the regulation of glucose absorption, insulin signaling, insulin secretion, and adipose deposition [61]. Additionally, they target a number of molecules that are involved in the regulation of various pathways such as stimulating the PLC/PKC and/or cAMP/PKA signaling pathways in order to improve β-cell proliferation, and promote insulin secretion [62], prevent cellular apoptosis via inhibition PI3K/Akt pathway [63], and lower hyperglycaemia through regulating hepatic glucose metabolism [61]. Furthermore, flavonoids have also been reported to prevent diabetes-associated microvascular complications such as protecting from diabetic retinopathy by improving the retinal SIRT-1 pathway, alleviating diabetic neuropathy via activation of Nrf-2/HO-1 and inhibition of nuclear factor K beta (NF-κB) as well as inhibition of advanced glycation end-products generation [64]. Findings from previous studies have also shown that tannins promote the utilization of carbohydrates by receptor cells to enhance glucose uptake via phosphorylation of the protein components involved in the signaling cascade of insulin-mediated glucose transport, including the insulin receptor (IR), Akt, and translocation of the glucose transporter 4 (GLUT 4) [65]. Previous *in vitro* and *in vivo* studies reported that monoterpenes, known as terpenoid, exert the antidiabetic effect by lowering the blood glucose levels, reducing TC, TG, and plasma glucose, as well as improving impaired renal function [66]. Additionally, it has also been reported that anthraquinones improve glucose tolerance, enhance glucose uptake in cells, and improve glycaemic levels via several pathways including stimulation of PPAR-γ, inhibition of α-glucosidase activity, and regulation of the AKT/GSK‐3β signaling pathway [67]. Hence, it may be concluded that the presence of these phytochemicals in EEAA is responsible for its insulinotropic and glucose-lowering effects. However, further research is certainly warranted to corroborate this hypothesis.

## Conclusion

To summarize, the current study has demonstrated that the anti-hyperglycaemic effects of EEAA bark are linked to decreased intestinal glucose absorption and increased tissue glucose utilization, which is facilitated by an increase in insulin release from clonal pancreatic β-cells and isolated mouse islets. In addition to that, the decrease in DPP-IV enzyme activity increased the amount of active GLP-1 (7-36) level in the systemic circulation. These effects might be attributed to the presence of various bioactive constituents such as flavonoids, tannins, terpenoids, and anthraquinones. As a result, we might speculate that *Acacia arabica* could be used as a dietary supplement as well as a possible source of oral antidiabetic agents to treat hyperglycaemia. However, further in-depth studies are needed to investigate the role of *Acacia arabica* and its marker compounds in the prevention and management of Type 2 diabetes in individuals.

## Institutional Animal Care

The Animal Welfare and Ethical Review Board (AWERB) at Ulster University approved the use of animals for research in May 2018. The UK Home Office issued project/personal license numbers PIL1822 and PPL2804 in May 2016 and February 2017, respectively, under which the experiments were conducted. All experiments were performed in the Biomedical and Behavioral Research Unit (BBRU) at Ulster University, Coleraine, U.K. in accordance with the UK Act 1986 and EU Directive 2010/63EU, and necessary measures were taken to make sure no animals were harmed throughout the course of the research. Samples of blood were obtained from the cut tail tips of live animals; they were not executed.

## Data Availability

All data are included in the manuscripts, and the identifiable participant information (PA) for the data collections is included in the Author Contribution section.

## Abbreviations

2 NBDG: 2-[N-(7-nitrobenz-2-oxa-1,3-diazol-4-yl) amino]-2-deoxy-D-glucose
AMPK: adenosine monophosphate protein kinase
cAMP: cyclic Adenosine monophosphate
CEDT: cellulose ester dialysis tube
DPP-IV: dipeptidyl peptidase-IV
EEAA: ethanol extract of *Acacia arabica*
FLIPR: fluorometric imaging plate reader
GIP: glucose-dependent insulinotropic polypeptide
GIT: gastrointestinal tract
GLP-1: glucagon-like peptide-1
GLUT-4: glucose transporter type 4
Gly-Pro-AMC: Gly-Pro-7-Amino-4-Methyl-Coumarin
GOD/PAP: glucose oxidase-phenol amino phenazone
GSK-3β: glycogen synthase kinase-3β
HFF: high fat-fed
HPLC: high-performance liquid chromatography
IRS: insulin receptor substrate
K_ATP_: adenosine triphosphate-sensitive potassium channel
KCl: potassium chloride
KRB: Krebs-Ringer bicarbonate
NF-kB: nuclear factor kappa B
NrF-2/HO-1: nuclear factor erythroid-2 related factor 2/heme oxygenase
PBS: phosphate-buffered saline
PI3K/Akt: phosphoinositide 3-kinase/protein kinase B
PKA: protein kinase A
PKB: protein kinase B
PLC/PKC: phospholipase C (PLC)/protein kinase C
PPAR-γ: peroxisome proliferator-activated receptor-γ
SIRT-1: Sirtuin 1
TC: total cholesterol
TG: triglycerides
